# Supplementary material for: Multiple New Strains of Amphidomataceae (Dinophyceae) from the North Atlantic Revealed a High Toxin Profile Variability of Azadinium spinosum and a New Non-Toxigenic Az. cf. spinosum
Source: Microorganisms. 2021 Jan 8;9(1):134. doi: 10.3390/microorganisms9010134 (PMC7826828; doi:10.3390/microorganisms9010134)
Supplement: Supplementary file 1 [file microorganisms-09-00134-s001.pdf]

Multiple new strains of Amphidomataceae (Dinophyceae) from the North Atlantic revealed high toxin profile variability of *Azadinium spinosum*, and a new non-toxigenic *Az.* cf. *spinosum*

Urban Tillmann[1*], Stephan Wietkamp[1], Haifeng Gu[2,3], Bernd Krock[1], Rafael Salas[4], Dave Clarke[4]

[1] Alfred Wegener Institute, Helmholtz Center for Polar and Marine Research, Am Handelshafen 12, D-27570 Bremerhaven, Germany
[2] Third Institute of Oceanography, Ministry of Natural Resources, Xiamen 361005, People's Republic of China
[3] School of Marine Sciences, Nanjing University of Information Science and Technology, Nanjing, 210044, China
[4] Marine Institute, Rinville, Oranmore, Co.Galway, HR91 R673, Republic of Ireland

*Corresponding author, e-mail address: urban.tillmann@awi.de

Supplementary material

Figure S1: ITS secondary structure of *Az. spinosum* and *Az.* cf. *spinosum*

Figure S2: LM and SEM of *Az. spinosum* ribotype B, strain 5-F3

Figure S3: LM and SEM of *Az. spinosum* ribotype B, strain 5-F7

Figure S4: LM and SEM of *Az. spinosum* ribotype B, strain 7-E4

Figure S5: LM and SEM of *Az.* cf. *spinosum*, strain 6-A1

Figure S6: SEM of plate details of *Az.* cf. *spinosum* strain 6-A1

Figure S7: LM and SEM of *Az.* cf. *spinosum*, strain 2-A3

Table S1: SMR transitions of AZA analysis

Table S2: Limits of detection of AZA analyses

Table S3: GenBank accession numbers of new strains and strains used for the phylogenetic analyses

Table S4: Strain compilation of *Az. spinosum*

Table S5: Strain compilation of *Am. languida*

Table S6: ITS based genetic distances of selected *Azadinium* strains

Table S7: qPCR specificity tests of new strains

Table S8: Summary statistics *Az. spinosum* ribotype A, AZA cell quota of selected strains

Table S9: Summary statistics *Az. spinosum* ribotype A, AZA-1/AZA-2 ratios of selected strains

Table S10: Summary statistics *Az. spinosum* ribotype A, AZA-1/AZA-33 ratios of selected strains

Table S11: Summary statistics *Az. spinosum* ribotype B, AZA cell quota of selected strains

Table S12: Summary statistics *Az. spinosum* ribotype B, AZA-11/AZA-51 ratios of selected strains.

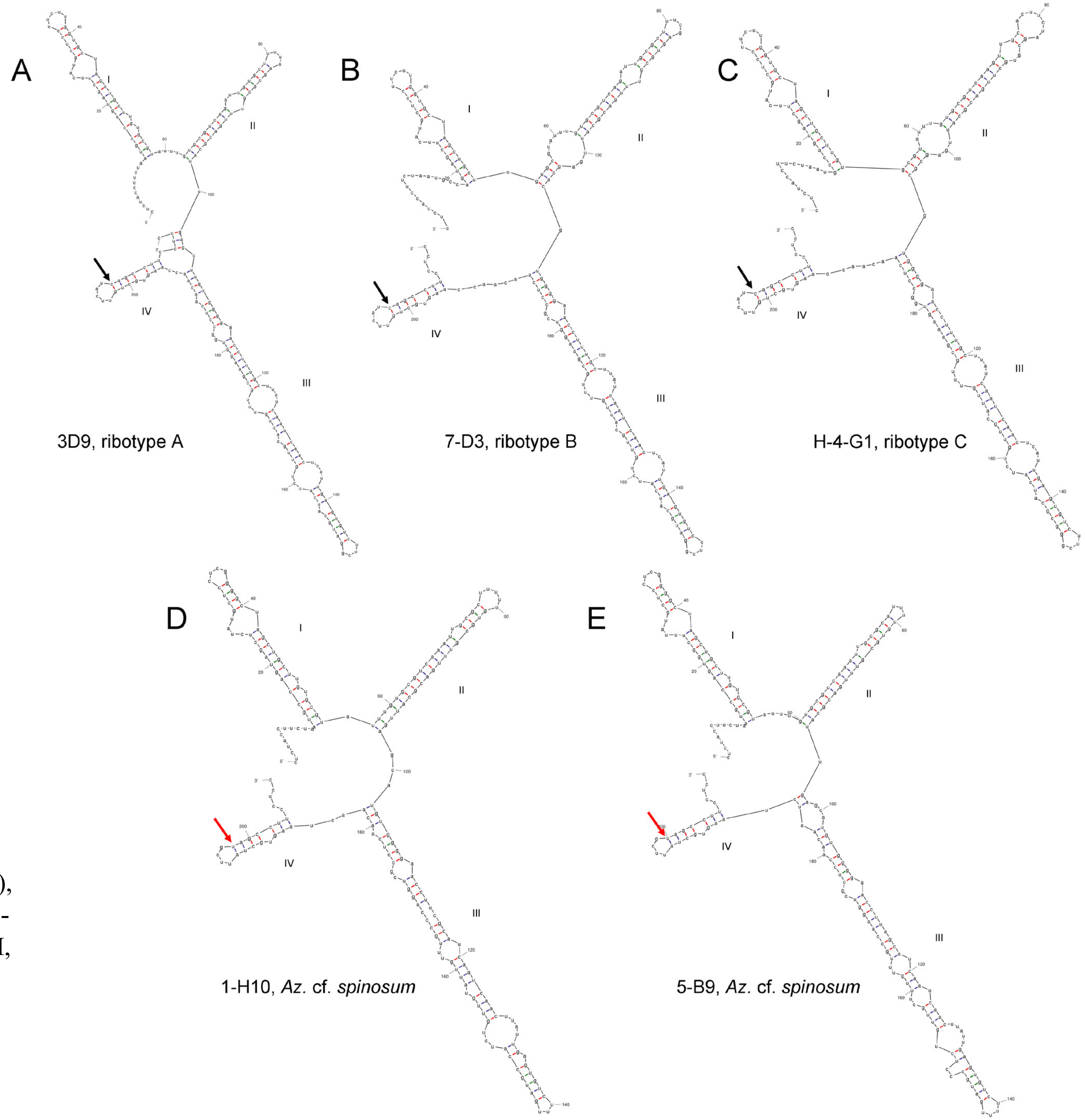

Figure S1. ITS2 secondary structure model of *Az. spinosum* strains 3D9 (A), 7-D3 (B), H-4-G1 (C) and *Az. cf. spinosum* strains 1-H10 (D), 5-B9 (E) showing four helices (I, II, III and IV) and a CBC in helix IV (arrows).

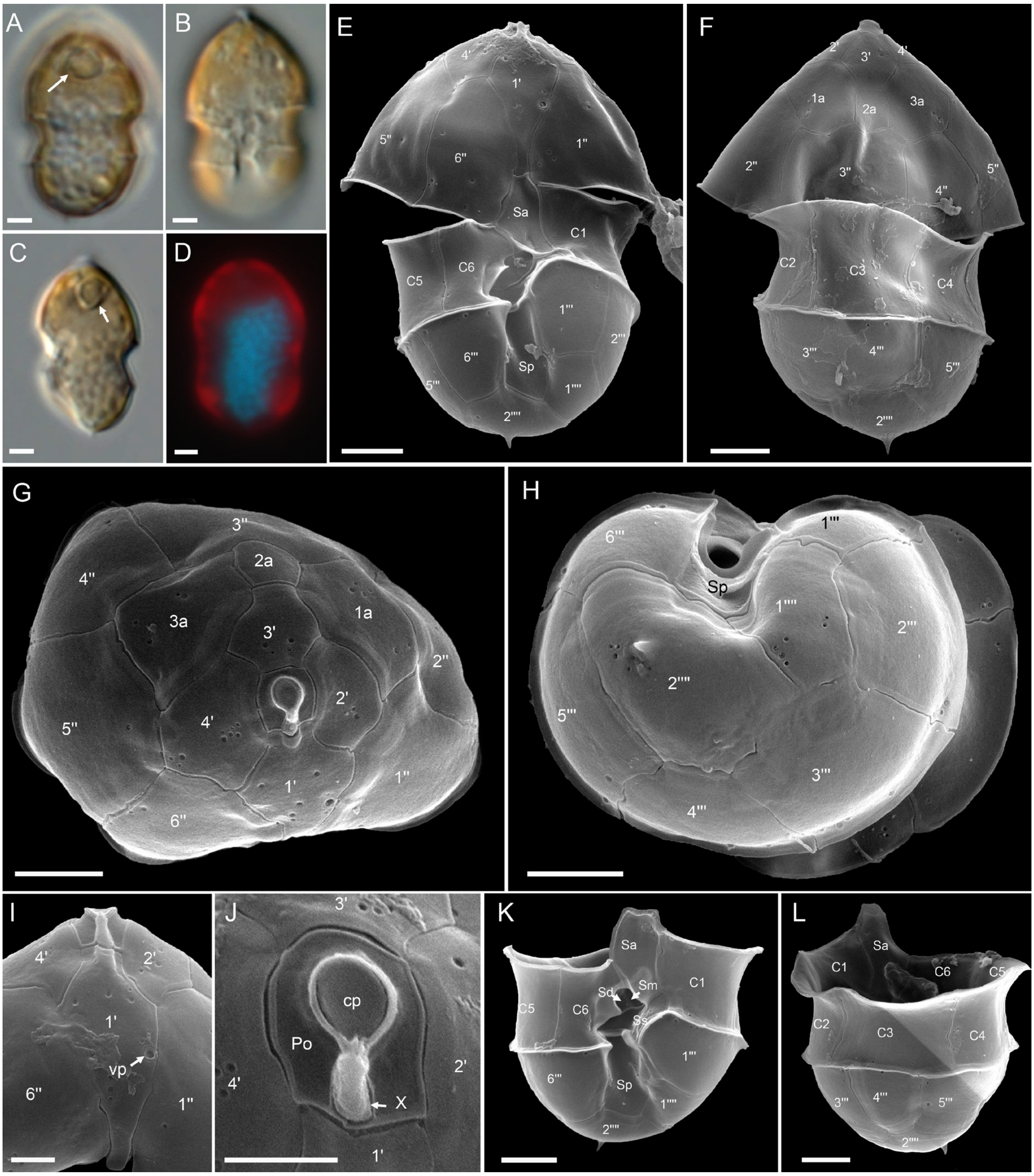

Figure S2. *Azadinium spinosum* ribotype B, strain 5-F3. (**A–D**) LM images to indicate general size and shape. Note the distinct pyrenoid in the episome (arrows in **A**, **C**). (**D**) Formalin fixed and DAPI-stained cells viewed with UV excitation to indicate shape and position if the nucleus. (**E–L**) SEM images of different thecae. (**E**) Ventral view. (**F**) Dorsal view. (**G**) Apical view of epithecal plates. (**H**) Antapical view of hypothecal plates. (**I**) Epitheca in ventral view, note the position of the ventral pore (vp). (**J**) Detailed view of the apical pore complex (APC). (**K**) Hypotheca and sulcal area in ventral view. (**L**) Hypotheca in dorsal view. Plate labels according the Kofoidian system. Po = pore plate; cp = cover plate; X = X-plate or canal plate; vp = ventral pore. Abbreviation of sulcal plates: Sa = anterior sulcal plate; Sp = posterior sulcal plate; Ss = left sulcal plate; Sm = median sulcal plate; Sd = right sulcal plate. Scale bars = 2 µm (**A–H**, **K**, **L**) or 1 µm (**I, J**).

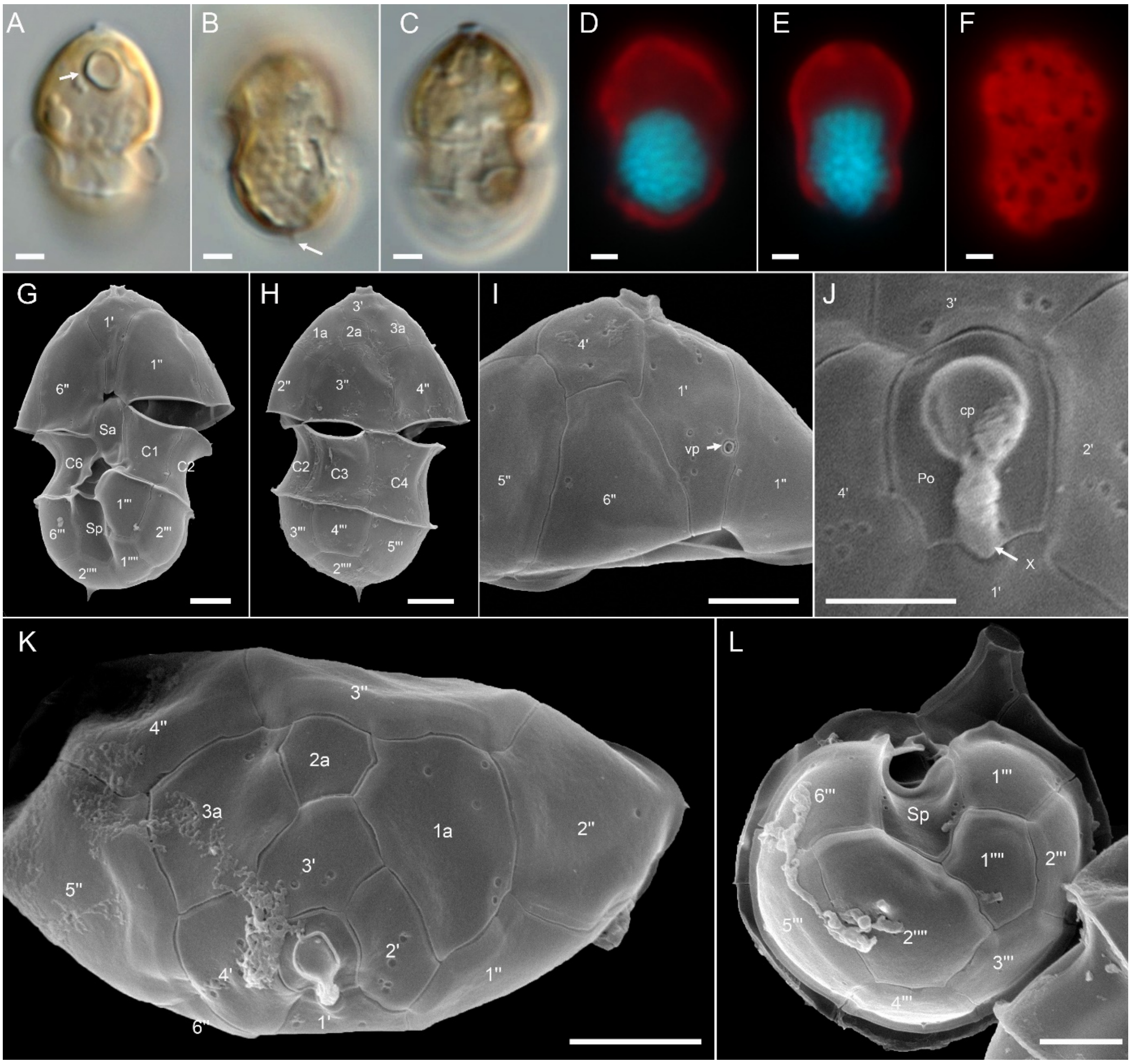

Figure S3. *Azadinium spinosum* ribotype B, strain 5-F7. (**A–F**) LM images to indicate general size and shape. Note the distinct pyrenoid in the episome (arrow in **A**) and the antapical spine (arrow in **B**). (**D–F**) Formalin fixed and DAPI-stained cells viewed with UV excitation to indicate shape and position of the nucleus (**D, E**) and of the chloroplast (**F**). (**G–L**) SEM images of different thecae. (**G**) Ventral view. (**H**) Dorsal view. (**I**) Epitheca in ventral view, note the position of the ventral pore (vp). (**J**) Detailed view of the apical pore complex (APC). (**K**) Apical view of epithecal plates. (**L**) Antapical view of hypothecal plates. Plate labels according the Kofoidian system. Po = pore plate; cp = cover plate; X = X-plate or canal plate; vp = ventral pore. Abbreviation of sulcal plates: Sa = anterior sulcal plate; Sp = posterior sulcal plate. Scale bars = 2 µm (**A–I, K, L**) or 1 µm (**J**).

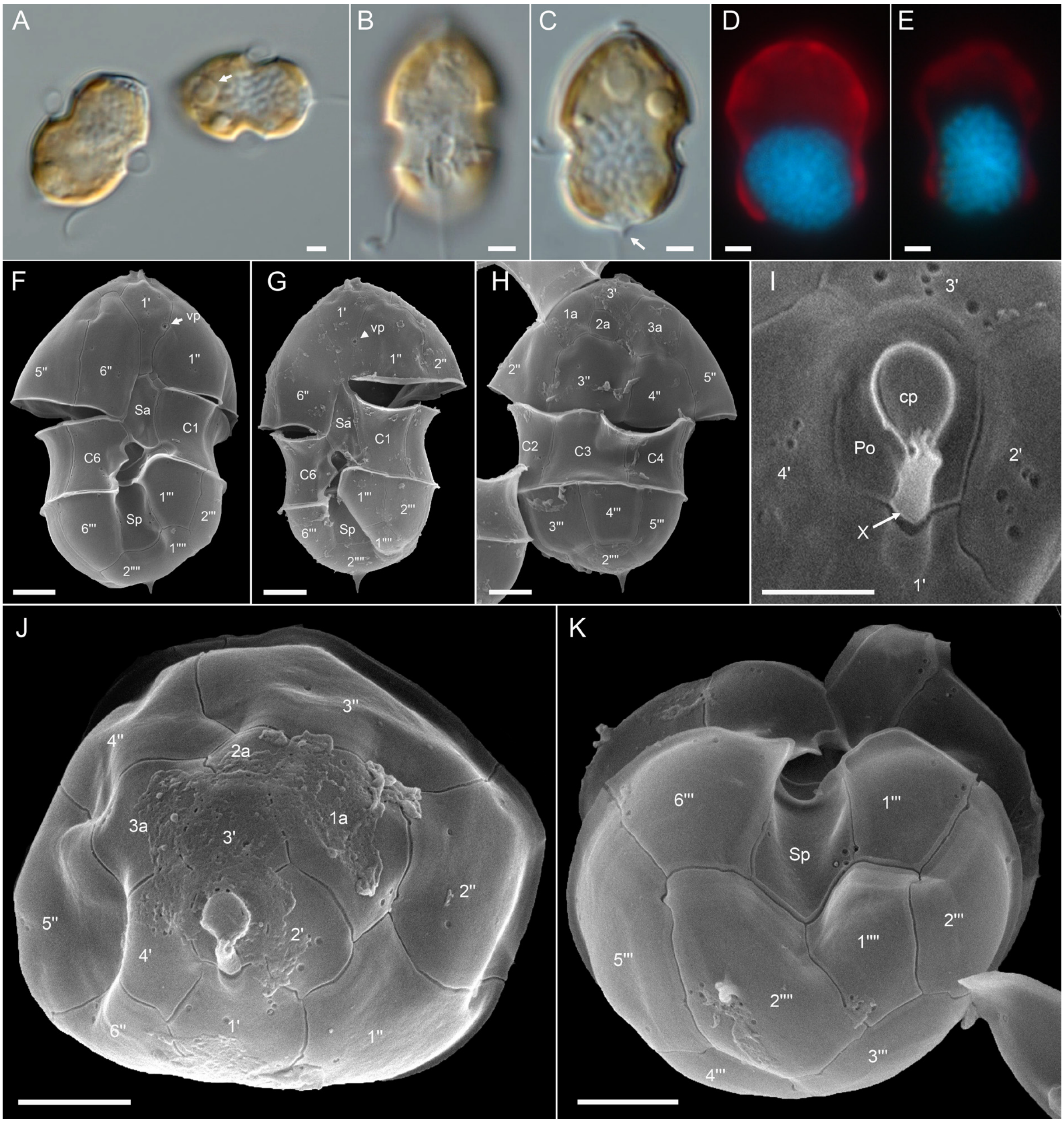

Figure S4. *Azadinium spinosum* ribotype B, strain 7-E4. (**A–E**) LM images to indicate general size and shape. Note the distinct pyrenoid in the episome (arrow in **A**) and the antapical spine (arrow in **C**). (**D, E**) Formalin fixed and DAPI-stained cells viewed with UV excitation to indicate shape and position of the nucleus. (**F–K**) SEM images of different thecae. (**F, G**) Ventral view. (**H**) Dorsal view. (**I**) Detailed view of the apical pore complex. (**J**) Apical view of epithecal plates. (**K**) Antapical view of hypothecal plates. Plate labels according the Kofoidian system. Po = pore plate; cp = cover plate; X = X-plate or canal plate; vp = ventral pore. Abbreviation of sulcal plates: Sa = anterior sulcal plate; Sp = posterior sulcal plate. Scale bars = 2 µm (**A–H, J, K**) or 1 µm (**I**).

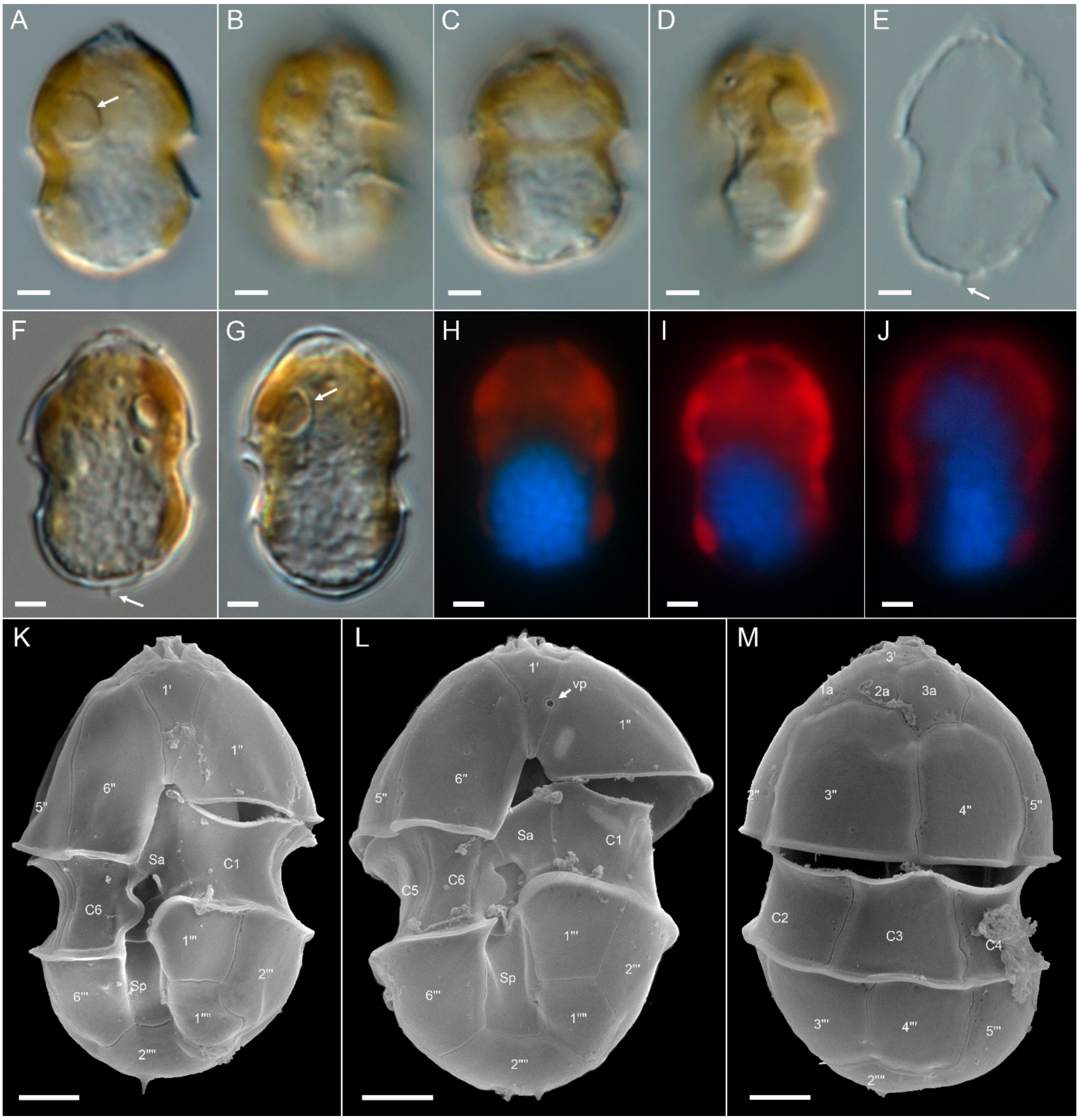

Figure S5. *Azadinium* cf. *spinosum* strain 6-A1. (**A–J**) LM images to indicate general size and shape. Note the distinct pyrenoid in the episome (arrows in **A, G**) and the antapical spine (arrow in **E, F**). (**H–J**) Formalin fixed and DAPI-stained cells viewed with UV excitation to indicate shape and position if the nucleus. (**K–M**) SEM images of different thecae. (**K, L**) Ventral view. (**M**) Dorsal view. Plate labels according the Kofoidian system. vp = ventral pore. Abbreviation of sulcal plates: Sa = anterior sulcal plate; Sp = posterior sulcal plate. Scale bars = 2 µm.

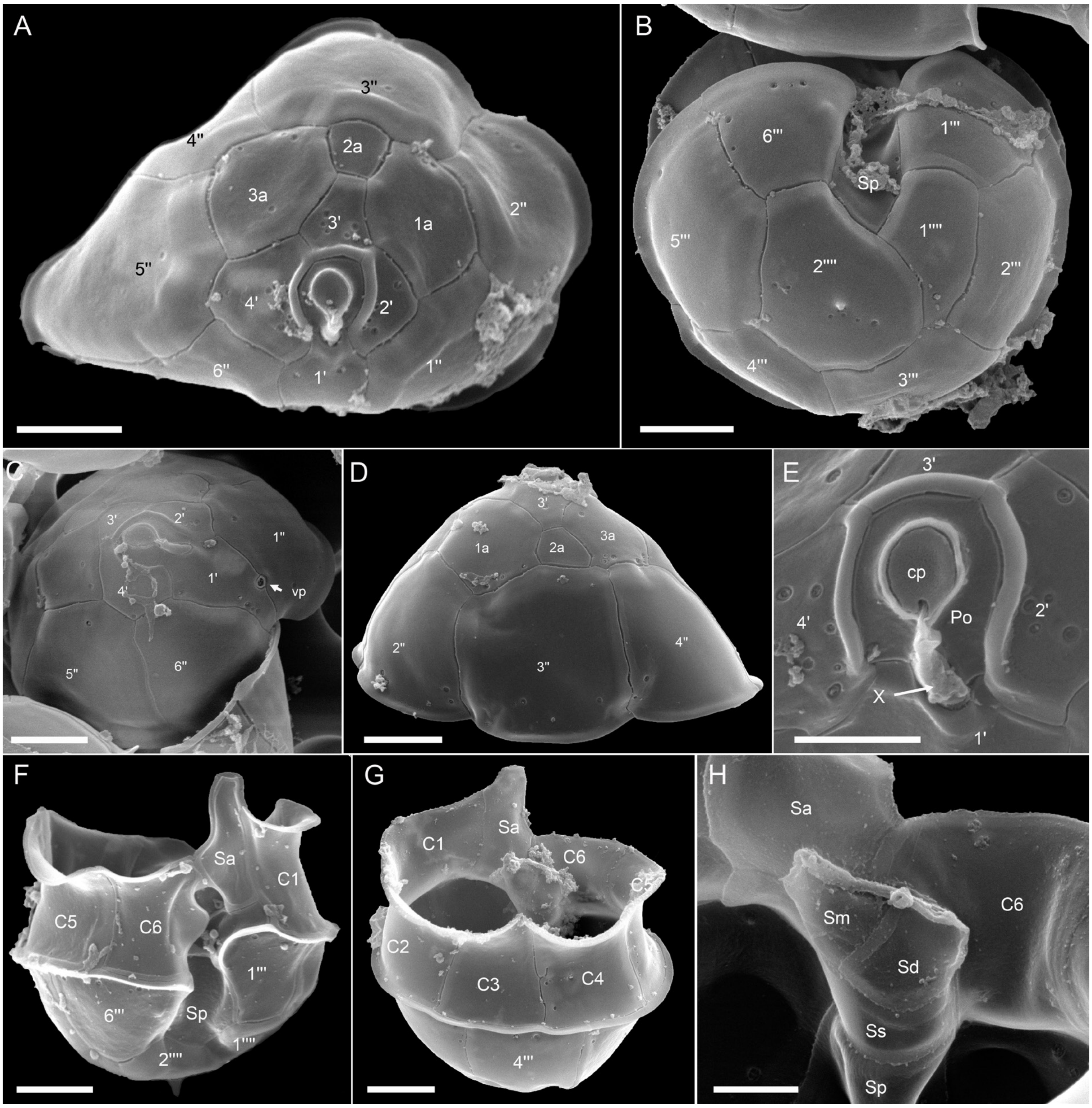

Figure S6. *Azadinium* cf. *spinosum*, strain 6-A1. SEM images of different cells. (**A**) Apical view of epithecal plates. (**B**) Antapical view of hypothecal plates. (**C**) Epitheca in apical/lateral view. (**D**) Epitheca in dorsal view. (**E**) Detailed view of the apical pore complex (APC). (**F**) Hypotheca and sulcal area in ventral view. (**G**) Hypotheca in dorsal view. (**H**) Internal view of sulcal plates. Plate labels according the Kofoidian system. Po = pore plate; cp = cover plate; X = X-plate or canal plate; vp = ventral pore. Abbreviation of sulcal plates: Sa = anterior sulcal plate; Sp = posterior sulcal plate; Ss = left sulcal plate; Sm = median sulcal plate; Sd = right sulcal plate. Scale bars = 2 µm (**A–D, F, G**) or 1 µm (**E, H**).

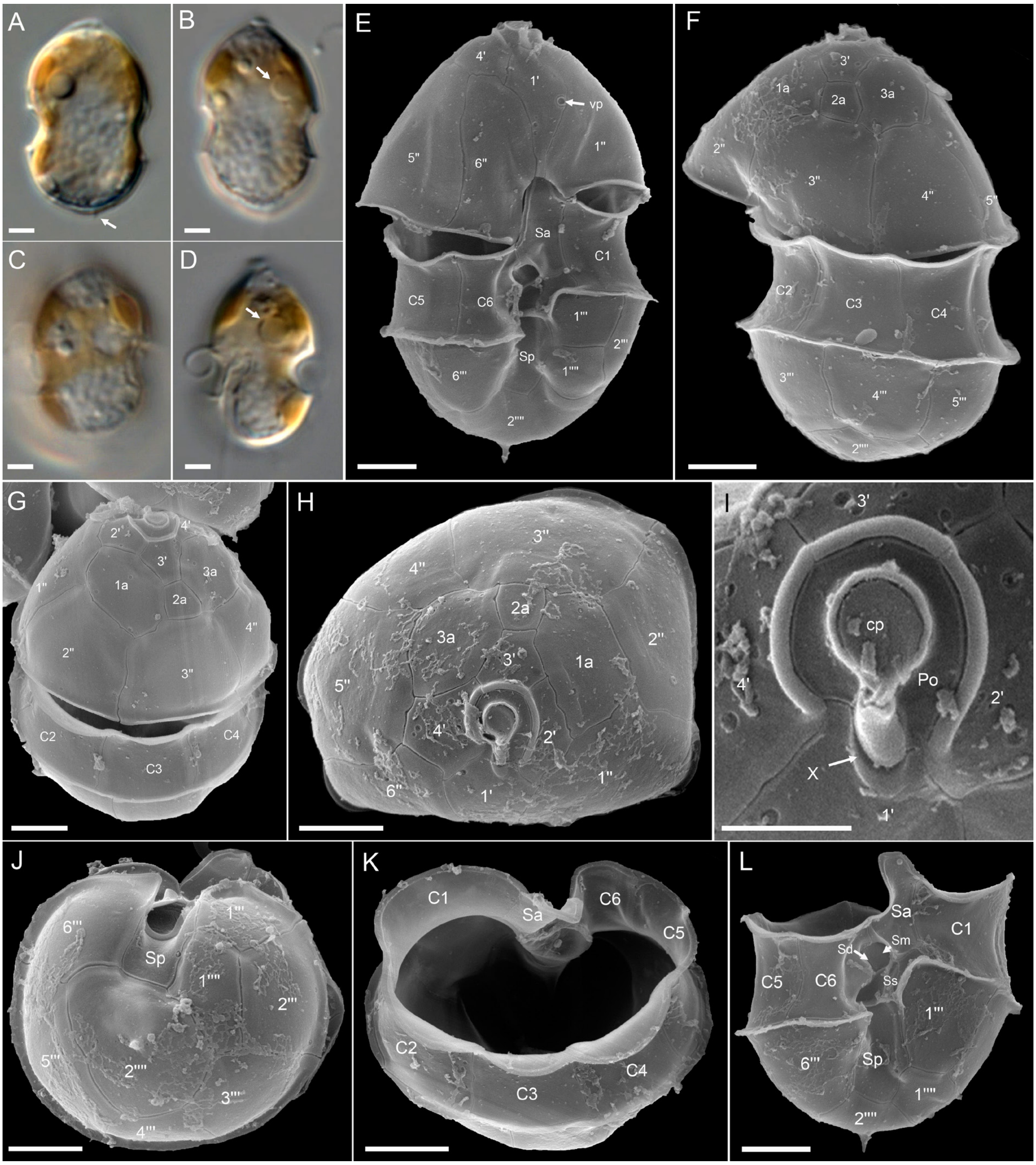

Figure S7. *Azadinium* cf. *spinosum,* strain 2-A3. (**A–D**) LM images to indicate general size and shape. Note the distinct pyrenoid in the episome (arrows in **B, D**) and the antapical spine (arrow in **A**). (**E–L**) SEM images of different thecae. (**E**) Ventral view. (**F**) Dorsal view. (**G**) Dorsal/apical view. (**H**) Apical view of epithecal plates. (**I**) Detailed view of the apical pore complex (APC). (**J**) Antapical view of hypothecal plates. (**K**) Hypotheca in apical/ dorsal view. (**L**) Hypotheca and sulcal area in ventral view. Plate labels according the Kofoidian system. Po = pore plate; cp = cover plate; X = X-plate or canal plate; vp = ventral pore. Abbreviation of sulcal plates: Sa = anterior sulcal plate; Sp = posterior sulcal plate; Ss = left sulcal plate; Sm = median sulcal plate; Sd = right sulcal plate. Scale bars = 2 µm (**A–H, J–L**) or 1 µm (**I**).

Suppl. Tables

Table S1: Selected reaction monitoring (SRM) transitions monitored for AZAs in the strains.

Mass transition	Toxin	Collision energy [V]
716>698	AZA-33	40
816>798	AZA-39, AZA-34	40
816>348	AZA-39	70
828>658	AZA-3, AZA-58	70
828>810	AZA-3, AZA-43	40
830>812	AZA-38, AZA-35	40
830>348	AZA-38	70
842>672	AZA-1	70
842>824	AZA-1, AZA-40	40
842>348	AZA-40	70
844>826	AZA-4, AZA-5, AZA-56	40
846>828	AZA-37	40
846>348	AZA-37	70
854>836	AZA-41	40
854>670	AZA-41	70
854>360	AZA-41	70
856>672	Me-AZA-1, AZA-2	70
856>838	AZA-2	40
858>840	AZA-7, AZA-8, AZA-9, AZA-10, AZA-36	40
858>348	AZA-36	70
860>842	AZA-59	40
868>850	AZA-55	40
868>362	AZA-55	70
870>852	Me-AZA-2, AZA-42, AZA-54, AZA-62	40
870>360	AZA-42	40
872>854	AZA-11, AZA-12	40
872>362	AZA-11, AZA-12	70
884>866	AZA-57	40
922>904	AZA-1 phosphate	40
936>918	AZA-2 phosphate	40
938>920	AZA-36 phosphate	40
940>842	AZA-59 phosphate	40
952>818	AZA-11 phosphate	40

Table S2: AZA analysis of "high biomass" samples of selected strains to calculate LOD for detecting known AZA (LOD SRM) and for detecting yet unknown AZA in precursor mode (LOD PREC).

Species	Strain	Profile	Pellet cells	LOD SRM known AZA (fg $cell^{-1}$)	LOD PREC unknown AZA (fg $cell^{-1}$)
Az. spinosum	5-C11	1, 2, 33	43,936,000	0.005	0.569
Az. spinosum	5-E4	1, 2, 33	46,559,000	0.001	0.054
Az. spinosum	6-G5	1, 2, 33	54,955,000	< 0.001	0.046
Az. spinosum	7-E10	1, 2, 33	22,899,250	0.002	0.076
Az. spinosum	7-F4	1, 2, 33	54,339,500	0.001	0.032
Az. spinosum	4-F8	1	46,056,000	0.001	0.054
Az. spinosum	6-G8	1, 2	33,134,100	0.001	0.098
Az. spinosum	2-C8	1, 33	90,041,000	< 0.001	0.028
Az. spinosum	5-F7	11, 51	3,571,000	0.012	0.488
Az. spinosum	7-D3	11, 51	6,980,250	0.002	0.358
Az. spinosum	8-B6	11, 51	7,672,250	0.002	0.326
Az. spinosum	5-F3	11, 51	2,880,300	0.015	0.606
Az. cf. spinosum	1-H10	-	35,917,200	0.001	0.049
Az. cf. spinosum	1-A3	-	90,713,000	< 0.001	0.019
Az. cf. spinosum	5-B9	-	25,535,400	0.002	0.068
Az. cf. spinosum	6-A1	-	27,979,800	0.002	0.062
Am. languida	5-F11	38, 39	1,122,150	0.024	2.230
Am. languida	8-D10	38, 39	5,967,720	0.007	0.292

Table S3. Species used for the molecular analyses based on SSU, ITS1–5.8S–ITS2 and LSU sequences, including strain designation, geographic origin and GenBank accession number. Only some selected new strains (marked with an asterisk (*)) were used to calculate the phylogenetic tree.

Species Name	Strain No.	Locality (Lat., Long)	GenBank No (SSU/ITS/LSU)	Ribotype	Reference
Azadinium caudatum var. caudatum	IFR1191 [IFR10-332, IFR10-330, IFR11-033]	North Atlantic, France (47°50'N, 03°57'W)	JQ247701 / JQ247700 / JQ247702		Nézan et al., 2012
Azadinium caudatum var. margalefii	AC1	North Atlantic, UK–Scotland (58°38'N, 03°36'W)	- / JQ247705 / JQ247709		Nézan et al., 2012
	IFR1190 [IFR10-020, IFR1140]	North Atlantic, France (47°50'N, 03°57'W)	JQ247707 / JQ247704 / JQ247708		Nézan et al., 2012
Azadinium concinnum	1C6	North Atlantic, Irminger Sea, (62°14'N, 37°27'W)	- / KJ481829 / KJ481830		Tillmann et al., 2014
Azadinium cuneatum	3D6	North Atlantic, Iceland (65°27'N, 24°39'W)	KJ481822 / KJ481823 / KJ481824		Tillmann et al., 2014
	965F5	Northeast Pacific, USA (47°49'N, 122°48'W)	- / KY404225 / KY404225		Kim et al., 2017
Azadinium dalianense	AZCH02	East China Sea, China (39°15'N, 122°36'E)	KF543360 / KF543358 / KF543359		Luo et al., 2013
	IFR-ADA-01C	North Atlantic, France (47°50'N, 3°57'W)	- / MF033117 / MF033126		Luo et al., 2017
	962B8	Northeast Pacific, USA (47°23'N, 122°58'W)	- / KY404222 (ITS+LSU)		Kim et al., 2017
	N-38-03	North Atlantic, Norway (62°20'N, 05°22'E)	LS974153 (SSU+ITS+LSU)		Tillmann et al., 2018a
	H-2-G7	South Atlantic, Argentina (41°06'S, 57°43'W)	- / MK405513 (ITS+LSU)		Tillmann et al., 2019
Azadinium dexteroporum	n.inf.	Mediterranean Sea, Gulf of Naples (40°49'N, 14°15'W)	- / KJ179946 / KJ179945		Percopo et al., 2013
	1-D12	North Atlantic, Irminger Sea (64°46'N, 29°57'W)	- / KR362889 / KR362887		Tillmann et al., 2015
	AZA-2-B1	Labrador Sea, Greenland (56°49'N, 52°13'W)	- / MK882968 / MK882952		Tillmann et al., 2020
Azadinium galwayense	35-R6	North Atlantic, Ireland (52° 1' N, 10° 46'W)	MT644698 / MT644671 / MT644682		Salas et al., in press
	35-R7	North Atlantic, Ireland (52° 1' N, 10° 46'W)	MT644699 / MT644672 / MT644683		Salas et al., in press
Azadinium obesum	2E10	North Atlantic, UK–Scotland (57°04'N, 02°30'W)	GQ914935 / FJ766093 / GQ914936		Tillmann et al., 2010
	LF-44-C03	North Atlantic, Denmark (54°33'N, 10°29'E)	- / MK612413 / MK613126		Wietkamp et al., 2019
	AZA-1G	Labrador Sea, Greenland (56°49'N, 52°13'W)	- / MK882967 / MK882951		Tillmann et al., 2020
	N-41-01	North Atlantic, Norway (62°51'N, 06°24'E)	LS974154 / - / LS974155		Tillmann et al., 2018a
Azadinium perforatum	AZA-2E	Labrador Sea, Greenland (56°49'N, 52°13'W)	MK883042 / MK882971 / MK882956		Tillmann et al., 2020
	AZA-2H	Labrador Sea, Greenland (56°49'N, 52°13'W)	MK883043 / MK882974 / MK882959		Tillmann et al., 2020
Azadinium perfusorium	2-D1	North Atlantic, Ireland (52° 1' N, 10° 46'W)	MT644700 / MT644673 / MT644684		Salas et al., in press
	5-B8	North Atlantic, Ireland (52° 1' N, 10° 46'W)	MT644701 / MT644674 / MT644685		Salas et al., in press
Azadinium polongum	SHETB2	North Atlantic, Shetland Islands (60°13'N, 01°00'W)	JX559886 (SSU+ITS+LSU)		Tillmann et al., 2012b
	N-47-01	North Atlantic, Norway (63°58'N, 08°37'E)	- / LS974156 (ITS+LSU)		Tillmann et al., 2018a
Azadinium poporum	UTHC5	North Atlantic, Denmark (56°15'N, 07°28'E)	HQ324897 / HQ324889 / HQ324893	A	Tillmann et al., 2011
	TIO256	Mediterranean, France (42°08'N, 09°32'E)	MF033112 / MF033116 / MF033123	A	Luo et al., 2017
	AZFC15	South China Sea, China (21°30'N, 108°14'E)	- / KC286569 / KC286550	B	Gu et al., 2013
	GM29	Gulf of Mexico, USA (29°19.50'N, 93°25.00'W)	- / KU686476 / KU686475	C	Luo et al., 2016
	TIO431	Mediterranean Sea, Greece (39°30'N, 20°15'E)	- / MH685508 / MH685486	A	Luo et al., 2018
Azadinium spinosum	3D9	North Atlantic, UK–Scotland (57°04'N, 02°30'W)	FJ217814 / FJ217816 / FJ217815	A	Tillmann et al., 2009
	SHETF6	North Atlantic, Shetland Islands (60°13'N, 01°00'W)	JX559885 (SSU+ITS+LSU)	A	Tillmann et al., 2012b

Tabl2 S3 cont.

Species Name	Strain No.	Locality (Lat., Long)	GenBank No (SSU/ITS/LSU)	Ribotype	Reference
Azadinium spinosum continued	UTHE2	North Atlantic, Denmark (56°15'N, 07°28'E)	HQ324900 / HQ324892 / HQ324896	A	Tillmann et al., 2011
	N-04-02	North Atlantic, Norway (58°15'N, 06°24'E)	LS974160 (SSU+ITS+LSU)	A	Tillmann et al., 2018a
	2-B5	North Atlantic, Ireland (52°47'N, 09°41'W)	- / MT791452 / MT791416	A	this study
	2-C8	North Atlantic, Ireland (52°01'N, 10°46'W)	- / MT791453 / MT791417	A	this study
	2-E2	North Atlantic, Ireland (53°37'N, 09°54'W)	- / MT791454 / MT791418	A	this study
	2-G10	North Atlantic, Ireland (53°37'N, 09°54'W)	- / MT791456 / MT791420	A	this study
	2-G4	North Atlantic, Ireland (53°37'N, 09°54'W)	- / MT791455 / MT791419	A	this study
	3-B4*	North Atlantic, Ireland (53°37'N, 09°54'W)	- / MT791457 / MT791421	A	this study
	3-E6	North Atlantic, Ireland (52°01'N, 10°46'W)	- / MT791458 / MT791422	A	this study
	4-C2	North Atlantic, Ireland (53°37'N, 09°54'W)	- / MT791459 / MT791423	A	this study
	4-D6	North Atlantic, Ireland (53°37'N, 09°54'W)	- / MT791460 / MT791424	A	this study
	4-E11	North Atlantic, Ireland (53°37'N, 09°54'W)	- / MT791461 / MT791425	A	this study
	4-E12	North Atlantic, Ireland (53°37'N, 09°54'W)	- / MT791462 / MT791426	A	this study
	4-F8	North Atlantic, Ireland (53°37'N, 09°54'W)	- / MT791463 / MT791427	A	this study
	4-G9*	North Atlantic, Ireland (52°01'N, 10°46'W)	- / MT791464 / MT791428	A	this study
	5-C11*	North Atlantic, Ireland (53°37'N, 09°54'W)	- / MT791465 / MT791429	A	this study
	5-C12	North Atlantic, Ireland (53°37'N, 09°54'W)	- / MT791466 / MT791430	A	this study
	5-E4	North Atlantic, Ireland (53°37'N, 09°54'W)	- / MT791467 / MT791431	A	this study
	6-A10*	North Atlantic, Ireland (53°37'N, 09°54'W)	- / MT791468 / MT791432	A	this study
	6-F12	North Atlantic, Ireland (53°37'N, 09°54'W)	- / MT791470 / MT791434	A	this study
	6-F8	North Atlantic, Ireland (53°37'N, 09°54'W)	- / MT791469 / MT791433	A	this study
	6-G5	North Atlantic, Ireland (53°37'N, 09°54'W)	- / MT791471 / MT791435	A	this study
	6-G8	North Atlantic, Ireland (53°37'N, 09°54'W)	- / MT791472 / MT791436	A	this study
	7-C1	North Atlantic, North Sea (55°21'N, 04°39'E)	- / MT791473 / MT791437	A	this study
	7-E10	North Atlantic, North Sea (55°21'N, 04°39'E)	- / - / MT791438	A	this study
	7-F4	North Atlantic, North Sea (55°21'N, 04°39'E)	- / MT791474 / MT791439	A	this study
	N-05-01	North Atlantic, Norway (58°22'N, 05°57'E)	LS974163 (SSU+ITS+LSU)	B	Tillmann et al., 2018a
	N-16-02	North Atlantic, Norway (60°52'N, 04°34'E)	LS974168 (SSU+ITS+LSU)	B	Tillmann et al., 2018a
	N-04-04	North Atlantic, Norway (58°15'N, 06°24'E)	LS974162 / - / LS974161	B	Tillmann et al., 2018a
	H-1-D11	South Atlantic, Argentina (41°06'S, 57°43'W)	MK405493 (SSU+ITS+LSU)	B	Tillmann et al., 2019
	5-F3	North Atlantic, North Sea (55°21'N, 04°39'E)	- / MT791475 / MT791440	B	this study
	5-F6	North Atlantic, North Sea (55°21'N, 04°39'E)	- / MT791476 / MT791441	B	this study
	5-F7	North Atlantic, North Sea (55°21'N, 04°39'E)	- / MT791477 / MT791442	B	this study
	5-G8*	North Atlantic, North Sea (55°21'N, 04°39'E)	- / MT791478 / MT791443	B	this study
	7-D3*	North Atlantic, North Sea (55°21'N, 04°39'E)	- / MT791479 / MT791444	B	this study
	7-E4	North Atlantic, North Sea (55°21'N, 04°39'E)	- / MT791480 / MT791445	B	this study
	8-B8	North Atlantic, North Sea (55°21'N, 04°39'E)	- / MT791481 / MT791446	B	this study
	H-4-G1	South Atlantic, Argentina (41°06'S, 57°43'W)	- / MK405497 (ITS+LSU)	C	Tillmann et al., 2019
	H-2-D8	South Atlantic, Argentina (41°06'S, 57°43'W)	- / MK405500 (ITS+LSU)	C	Tillmann et al., 2019

Tabl2 S3 cont.

Species Name	Strain No.	Locality (Lat., Long)	GenBank No (SSU/ITS/LSU)	Ribotype	Reference
Azadinium spinosum continued	H-4-F9	South Atlantic, Argentina (41°06’S, 57°43’W)	- / MK405510 (ITS+LSU)	C	Tillmann et al., 2019
	H-4-G9	South Atlantic, Argentina (41°06’S, 57°43’W)	- / MK405512 (ITS+LSU)	C	Tillmann et al., 2019
	H-4-E9	South Atlantic, Argentina (41°06’S, 57°43’W)	- / MK405509 (ITS+LSU)	C	Tillmann et al., 2019
	H-4-E6	South Atlantic, Argentina (41°06’S, 57°43’W)	- / MK405508 (ITS+LSU)	C	Tillmann et al., 2019
Azadinium cf. *spinosum*	1-H10*	North Atlantic, Ireland (52°01’N, 10°46’W)	MT791401 / MT791482 / MT791447		this study
	2-A3*	North Atlantic, Ireland (52°01’N, 10°46’W)	MT791402 / MT791483 / MT791448		this study
	5-B9*	North Atlantic, Ireland (52°01’N, 10°46’W)	MT791403 / MT791484 / MT791449		this study
	5-D3*	North Atlantic, Ireland (52°01’N, 10°46’W)	MT791404 / - / MT791450		this study
	6-A1*	North Atlantic, Ireland (52°01’N, 10°46’W)	MT791405 / MT791485 / MT791451		this study
Azadinium trinitatum	4A8	North Atlantic, Iceland (64°43’N, 24°02’W)	KJ481808 / KJ481809 / KJ481810		Tillmann et al., 2014
	A2D11	North Atlantic, Iceland (64°43’N, 24°02’W)	KJ481803 / KJ481806 / KJ481807		Tillmann et al., 2014
	N-39-04	North Atlantic, Norway (62°28’N, 05°46’E)	LS974170 (SSU+ITS+LSU)		Tillmann et al., 2018a
	AZA-2F	Labrador Sea, Greenland (56°49’N, 52°13’W)	- / MK882972 / MK882957		Tillmann et al., 2020
	AZA-Z-E10	Labrador Sea, Greenland (56°49’N, 52°13’W)	- / MK882979 / MK882964		Tillmann et al., 2020
Azadinium zhuanum	TIO205	East China Sea, China (27°28’N, 121°03’E)	MF033110 / MF033114 / MF033120		Luo et al., 2017
	TIO213	East China Sea, China (27°28’N, 121°03’E)	MF033111 / MF033115 / MF033121		Luo et al., 2017
Azadinium cf. *zhuanum*	32-R1	North Atlantic, Ireland (51° 32'N, 10° 0'W)	- / MT644679 / MT644695		Salas et al., in press
Amphidoma languida	SM1	North Atlantic, Ireland (51°39’N, 09°35’E)	JN615412 / JQ247699 / JN615413		Tillmann et al., 2012a
	2A11	North Atlantic, Iceland (65°27’N, 24°39’W)	KR362880 / KR362882 / KR362885		Tillmann et al., 2015
	AND-A0920	eastern North Atlantic, Spain, (37°11’N, 07°02’W)	KX671042 / KX671038 / KX671043		Tillmann et al., 2017
	N-33-01	North Atlantic, Norway (61°07’N, 04°28’E)	LS974133 / - / LS974132		Tillmann et al., 2018a
	N-40-04	North Atlantic, Norway (62°43’N, 06°03’E)	LS974145 / - / LS974144		Tillmann et al., 2018a
	5-F10*	North Atlantic, North Sea (55°21’N, 04°39’E)	- / - / MT791406		this study
	5-F11	North Atlantic, North Sea (55°21’N, 04°39’E)	- / - / MT791407		this study
	6-D11*	North Atlantic, North Sea (55°21’N, 04°39’E)	- / - / MT791408		this study
	7-F8	North Atlantic, North Sea (55°21’N, 04°39’E)	- / - / MT791409		this study
	7-G4	North Atlantic, North Sea (55°21’N, 04°39’E)	- / - / MT791410		this study
	7-G6	North Atlantic, North Sea (55°21’N, 04°39’E)	- / - / MT791411		this study
	7-H4	North Atlantic, North Sea (55°21’N, 04°39’E)	- / - / MT791412		this study
	7-H5	North Atlantic, North Sea (55°21’N, 04°39’E)	- / - / MT791413		this study
	8-C4	North Atlantic, North Sea (55°21’N, 04°39’E)	- / - / MT791414		this study
	8-D10	North Atlantic, North Sea (55°21’N, 04°39’E)	- / - / MT791415		this study
Amphidoma parvula	H-1E9	Southwest Atlantic, Argentina (41°5.6′S, 57°43.2′W)	KY996792		Tillmann et al., 2018b

References Table S3

Gu, H., Luo, Z., Krock, B., Witt, M., Tillmann, U., 2013. Morphology, phylogeny and azaspiracid profile of *Azadinium poporum* (Dinophyceae) from the China Sea. Harmful Algae **21-22**, 64-75.

Kim, J.H., Tillmann, U., Adams, N.G., Krock, B., Stutts, W.L., Deeds, J.R., Han, M.S., Trainer, V.L., 2017. Identification of *Azadinium* species and a new azaspiracid from *Azadinium poporum* in Puget Sound, Washington State, USA. Harmful Algae **68**, 152-167.

Luo, Z., Gu, H., Krock, B., Tillmann, U., 2013. *Azadinium dalianense*, a new dinoflagellate species from the Yellow Sea, China. Phycologia **52**(6), 625-636.

Luo, Z., Krock, B., Giannakourou, A., Venetsanopoulou, A., Pagou, K., Tillmann, U., Gu, H., 2018. Sympatric occurrence of two *Azadinium poporum* ribotypes in the Eastern Mediterranean Sea. Harmful Algae **78**, 75-85.

Luo, Z.H., Krock, B., Mertens, K.N., Nézan, E., Chomérat, N., Billen, G., Tillmann, U., Gu, H., 2017. Adding new pieces to the *Azadinium* (Dinophyceae) diversity and biogeography puzzle: Non-toxigenic *Azadinium zhuanum* sp. nov. from China, toxigenic *A. poporum* from the Mediterranean, and a non-toxigenic *A. dalianense* from the French Atlantic. Harmful Algae **66**, 65-78.

Luo, Z.H., Krock, B., Mertens, K.N., Price, A.M., Turner, R.E., Rabalais, N.N., Gu, H.F., 2016. Morphology, molecular phylogeny and azaspiracid profile of *Azadinium poporum* (Dinophyceae) from the Gulf of Mexico. Harmful Algae **55**, 56-65.

Nézan, E., Tillmann, U., Bilien, G., Boulben, S., Cheze, K., Zentz, F., Salas, R., Chomerat, N., 2012. Taxonomic revision of the dinoflagellate *Amphidoma caudata*: Transfer to the genus *Azadinium* (Dinophyceae) and proposal of two varieties, based on morphological and molecular phylogenetic analyses. J. Phycol. **48**(4), 925-939.

Percopo, I., Siano, R., Rossi, R., Soprano, V., Sarno, D., Zingone, A., 2013. A new potentially toxic *Azadinium* species (Dinophyceae) from the Mediterranean Sea, *A. dexteroporum* sp. nov. J. Phycol. **49**(5), 950-966.

Salas, R., Tillmann, U., Gu, H. Wietkamp, S., Krock, B., Clarke, D., 2021. Morphological and molecular characterization of multiple new *Azadinium* strains revealed a high diversity of non-toxigenic species of Amphidomataceae (Dinophyceae) including two new *Azadinium* species in Irish waters, North East Atlantic. Phycological Research, doi:10.1111/pre.12448

Tillmann, U., Elbrächter, M., Krock, B., John, U., Cembella, A., 2009. *Azadinium spinosum* gen. et sp. nov. (Dinophyceae) identified as a primary producer of azaspiracid toxins. Eur. J. Phycol. **44**(1), 63-79.

Tillmann, U., Elbrächter, M., John, U., Krock, B., Cembella, A., 2010. *Azadinium obesum* (Dinophyceae), a new nontoxic species in the genus that can produce azaspiracid toxins. Phycologia **49**(2), 169-182.

Tillmann, U., Elbrächter, M., John, U., Krock, B., 2011. A new non-toxic species in the dinoflagellate genus *Azadinium*: *A. poporum* sp. nov. Eur. J. Phycol. **46**(1), 74-87.

Tillmann, U., Salas, R., Gottschling, M., Krock, B., O'Driscoll, D., Elbrächter, M., 2012a. *Amphidoma languida* sp. nov. (Dinophyceae) reveals a close relationship between *Amphidoma* and *Azadinium*. Protist **163**(5), 701-719.

Tillmann, U., Soehner, S., Nézan, E., Krock, B., 2012b. First record of the genus *Azadinium* (Dinophyceae) from the Shetland Islands, including the description of *Azadinium polongum* sp. nov. Harmful Algae **20**, 142-155.

Tillmann, U., Gottschling, M., Nezan, E., Krock, B., Bilien, G., 2014a. Morphological and molecular characterization of three new *Azadinium* species (Amphidomataceae, dinophyceae) from the Irminger sea. Protist **165**(4), 417-444.

Tillmann, U., Krock, B., Taylor, B.B., 2014b. *Azadinium caudatum* var. *margalefii*, a poorly known member of the toxigenic genus *Azadinium* (Dinophyceae). Mar. Biol. Res. **10**(10), 941-956.

Tillmann, U., Gottschling, M., Nézan, E., Krock, B., 2015. First records of *Amphidoma languida* and *Azadinium dexteroporum* (Amphidomataceae, Dinophyceae) from the Irminger Sea off Iceland. Mar. Biodivers. Rec. **8**, 1-11.

Tillmann, U., Borel, C.M., Barrera, F., Lara, R., Krock, B., Almandoz, G.O., Witt, M., Trefault, N., 2016. *Azadinium poporum* from the Argentine Continental Shelf, Southwestern Atlantic, produces azaspiracid-2 and azaspiracid-2 phosphate. Harmful Algae **51**, 40-55.

Tillmann, U., Jaén, D., Fernández, L., Gottschling, M., Witt, M., Blanco, J., Krock, B., 2017a. *Amphidoma languida* (Amphidomatacea, Dinophyceae) with a novel azaspiracid toxin profile identified as the cause of molluscan contamination at the Atlantic coast of southern Spain. Harmful Algae **62**, 113-126.

Tillmann, U., Trefault, N., Krock, B., Parada-Pozo, G., De la Iglesia, R., Vasquez, M., 2017b. Identification of *Azadinium poporum* (Dinophyceae) in the Southeast Pacific: morphology, molecular phylogeny, and azaspiracid profile characterization. J. Plankton Res. **39**(2), 350-367.

Tillmann, U., Edvardsen, B., Krock, B., Smith, K.F., Paterson, R.F., Voß, D., 2018a. Diversity, distribution, and azaspiracids of Amphidomataceae (Dinophyceae) along the Norwegian coast. Harmful Algae **80**, 15-34.

Tillmann, U., Gottschling, M., Guinder, V., Krock, B., 2018b. *Amphidoma parvula* (Amphidomataceae), a new planktonic dinophyte from the Argentine Sea. Eur. J. Phycol. **53**(1), 14-28.

Tillmann, U., S. Wietkamp, Krock, B., Tillmann, A., Voss, D., Gu, H., 2020. Amphidomataceae (Dinophyceae) in the western Greenland area, including description of *Azadinium perforatum* sp. nov. Phycologia **59**(1), 63-88.

Wietkamp, S., Krock, B., Gu, H., Voß, D., Klemm, K. Tillmann, U., 2019. Occurrence and distribution of Amphidomataceae (Dinophyceae) in Danish coastal waters of the North Sea, the Limfjord and the Kattegat/Belt area. Harmful Algae, **88,** 101637.

Table S4: Compilation of strains of *Az. spinosum* and *Az.* cf. *spinosum* obtained in this study

Species	Strain	Origin Station	Length (µm) Mean ± SD Min-max	Width (µm) Mean ± SD Min-max	l/w ratio Mean ± SD	N	Morphological analysis	Sequence data	AZA profile
Az. spinosum	4-C2	45	**14.3**±1.1 12.0-16.8	**9.6**±0.8 7.8-11.2	**1.50**±0.07	55	LM SEM	LSU, ITS	1, 2, 33
Az. spinosum	2-E2	45	**14.7**±1.2 12.1-17.1	**9.3**±0.7 8.1-10.6	**1.57**±0.09	52	LM SEM	LSU, ITS	1, 2, 33
Az. spinosum	4-E12	45	**15.5**±1.1 13.3-17.6	**10.1**±0.8 8.4-11.8	**1.54**±0.08	50	LM SEM	LSU, ITS	1, 2, 33
Az. spinosum	5-C12	45	**13.8**±1.0 11.2-16.3	**9.3**±0.8 7.9-11.7	**1.49**±0.09	64	LM SEM	LSU, ITS	1, 2, 33
Az. spinosum	7-E10	71	**15.0**±1.2 11.8-18.2	**9.4**±0.8 8.2-11.3	**1.61**±0.09	54	LM SEM	LSU	1, 2, 33
Az. spinosum	2-G4	45	-	-	-	-	LM SEM	LSU, ITS	1, 2, 33
Az. spinosum	5-E4	45	-	-	-	-	LM SEM	LSU, ITS	1, 2, 33
Az. spinosum	7-C1	71	-	-	-	-	LM SEM	LSU, ITS	1, 2, 33
Az. spinosum	5-C11	45	-	-	-	-	LM SEM	LSU, ITS	1, 2, 33
Az. spinosum	7-F4	71	-	-	-	-	LM SEM	LSU, ITS	1, 2, 33
Az. spinosum	2-B5	38	-	-	-	-	LM	LSU, ITS	1, 2, 33
Az. spinosum	6-F8	45	-	-	-	-	LM	LSU, ITS	1, 2, 33
Az. spinosum	6-G5	45	-	-	-	-	LM	LSU, ITS	1, 2, 33
Az. spinosum	4-D3	45	**14.5**±0.6 13.4-16.0	**9.3**±0.5 8.3-10.9	**1.56**±0.07	52	LM SEM	-	1, 2, 33
Az. spinosum	35-R1	35	**15.2**±1.3 12.9-18.3	**9.95**±1.2 7.9-12.9	**1.53**±0.11	50	LM SEM	-	1, 2, 33
Az. spinosum	35-R5	35	**15.2**±1.3 12.9-18.3	**9.95**±1.2 7.9-12.9	**1.53**±0.11	50	LM SEM	-	1, 2, 33
Az. spinosum	2-E3	45	-	-	-	-	LM SEM	-	1, 2, 33
Az. spinosum	2-E9	45	-	-	-	-	LM SEM	-	1, 2, 33
Az. spinosum	2-E5	45	-	-	-	-	LM	-	1, 2, 33
Az. spinosum	2-C12	35	-	-	-	-	LM	-	1, 2, 33
Az. spinosum	4-F10	35	-	-	-	-	LM	-	1, 2, 33
Az. spinosum	4-G8	35	-	-	-	-	LM	-	1, 2, 33
Az. spinosum	4-H2	45	-	-	-	-	LM	-	1, 2, 33
Az. spinosum	6-B10	35	-	-	-	-	LM	-	1, 2, 33
Az. spinosum	3-E12	35	-	-	-	-	-	-	1, 2, 33
Az. spinosum	2-E4	45	-	-	-	-	-	-	1, 2, 33
Az. spinosum	2-G8	45	-	-	-	-	-	-	1, 2, 33
Az. spinosum	2-H7	45	-	-	-	-	-	-	1, 2, 33
Az. spinosum	5-D7	35	-	-	-	-	-	-	1, 2, 33
Az. spinosum	5-E10	45	-	-	-	-	-	-	1, 2, 33
Az. spinosum	6-C4	22	-	-	-	-	-	-	1, 2, 33
Az. spinosum	6-F9	45	-	-	-	-	-	-	1, 2, 33
Az. spinosum	4-E11	45	**14.3**±0.9 12.8-16.4	**9.3**±0.7 7.8-10.7	**1.55**±0.08	50	LM SEM	LSU, ITS	1
Az. spinosum	4-F8	45	**15.6**±1.1 13.6-17.6	**10.0**±0.9 8.2-11.9	**1.56**±0.07	51	LM SEM	LSU, ITS	1
Az. spinosum	3-E6	35	**14.5**±0.9 12.9-16.8	**9.2**±0.5 8.1-10.3	**1.58**±0.08	51	LM SEM	LSU, ITS	1
Az. spinosum	3-B4	45	**14.7**±1.0 12.5-16.7	**9.5**±0.7 7.8-11.3	**1.54**±0.08	51	LM SEM	LSU, ITS	1
Az. spinosum	4-C9	45	**15.2**±1.0 13.7-17.7	**10.0**±0.7 8.4-11.5	**1.51**±0.07	51	LM SEM	-	1

Species	Strain	Origin Station	Length (µm) Mean ± SD Min-max	Width (µm) Mean ± SD Min-max	l/w ratio Mean ± SD	N	Morphological analysis	Sequence data	AZA profile
Az. spinosum	2-E6	45	-	-	-	-	LM SEM	-	1
Az. spinosum	6-F3	45	-	-	-	-	LM	-	1
Az. spinosum	6-F5	45	-	-	-	-	LM	-	1
Az. spinosum	6-B3	45	-	-	-	-	-	-	1
Az. spinosum	6-G19	45	-	-	-	-	-	-	1
Az. spinosum	6-G8	45	**15.1**±1.2 12.2-18.7	**9.9**±0.8 8.0-11.8	**1.53**±0.07	49	LM SEM	LSU, ITS	1, 2
Az. spinosum	4-D6	45	**14.8**±0.8 12.8-16.2	**9.7**±0.8 8.4-11.4	**1.52**±0.07	50	LM SEM	LSU, ITS	1, 2
Az. spinosum	4-G9	35	**14.0**±1.0 12.1-16.7	**9.2**±0.6 7.9-10.6	**1.52**±0.08	50	LM SEM	LSU, ITS	1, 2
Az. spinosum	2-G10	45	**14.1**±0.9 12.8-16.6	**9.1**±0.7 8.1-11.0	**1.55**±0.05	53	LM SEM	LSU, ITS	1, 2
Az. spinosum	2-F7	45	**14.5**±0.9 11.9-16.1	**9.6**±0.8 7.9-11.1	**1.51**±0.09	50	LM SEM	-	1, 2
Az. spinosum	1-D10	35	-	-	-	-	LM	-	1, 2
Az. spinosum	6-G1	45	-	-	-	-	LM	-	1, 2
Az. spinosum	2-E7	45	-	-	-	-	LM	-	1, 2
Az. spinosum	2-G2	45	-	-	-	-	-	-	1, 2
Az. spinosum	3-E2	35	-	-	-	-	-	-	1, 2
Az. spinosum	4-F1	45	-	-	-	-	-	-	1, 2
Az. spinosum	5-D1	45	-	-	-	-	-	-	1, 2
Az. spinosum	6-A6	32	-	-	-	-	-	-	1, 2
Az. spinosum	2-C8	35	**14.5**±0.9 11.9-16.5	**9.7**±0.6 8.2-10.7	**1.50**±0.07	53	LM SEM	LSU, ITS	1, 33
Az. spinosum	6-A10	45	**14.3**±1.0 12.6-16.9	**9.1**±0.7 7.8-10.9	**1.57**±0.07	52	LM SEM	LSU, ITS	1, 33
Az. spinosum	6-F12	45	**14.2**±1.0 12.5-16.5	**9.0**±0.6 7.7-10.4	**1.58**±0.09	51	LM SEM	LSU, ITS	1, 33
Az. spinosum	5-E2	45	-	-	-	-	LM	-	1, 33
Az. spinosum	6-F1	45	-	-	-	-	LM	-	1, 33
Az. spinosum	8-B8	71	**16.8**±0.9 14.6-18.8	**10.5**±0.7 9.2-12.0	**1.60**±0.07	50	LM SEM	LSU, ITS	11, 51
Az. spinosum	7-D3	71	**16.2**±1.1 13.7-18.7	**10.0**±0.8 8.6-11.8	**1.62**±0.09	52	LM SEM	LSU, ITS	11, 51
Az. spinosum	5-F6	71	**15.8**±0.8 13.3-16.7	**9.7**±0.7 8.3-11.6	**1.52**±0.07	54	LM SEM	LSU, ITS	11, 51
Az. spinosum	5-F7	71	**15.2**±1.1 13.4-17.9	**10.0**±0.8 8.8-12.2	**1.52**±0.07	52	LM SEM	LSU, ITS	11, 51
Az. spinosum	5-G8	71	-	-	-	-	LM SEM	LSU, ITS	11, 51
Az. spinosum	7-E4	71	-	-	-	-	LM SEM	LSU, ITS	11, 51
Az. spinosum	5-F3	71	-	-	-	-	LM SEM	LSU, ITS	11, 51
Az. cf. *spinosum*	1-H10	35	**15.0**±0.9 13.4–17.6	**9.5**±0.5 8.3–10.6	**1.58**±0.08	31	LM SEM	SSU, LSU, ITS	no AZA
Az. cf. *spinosum*	2-A3	35	**15.1**±0.9 13.1–16.8	**9.8**±0.7 8.3–10.8	**1.55**±0.07	56	LM SEM	SSU, LSU, ITS	no AZA
Az. cf. *spinosum*	6-A1	35	**16.6**±0.9 14.9–18.7	**10.7**±0.8 9.5–12.9	**1.56**±0.08	54	LM SEM	SSU, LSU, ITS	no AZA
Az. cf. *spinosum*	5-B9	35	**14.9**±0.8 12.9–16.9	**10.0**±0.7 8.1–11.5	**1.49**±0.06	54	LM SEM	SSU, LSU, ITS	no AZA
Az. cf. *spinosum*	5-D3	35	-	-	-	-	LM SEM	SSU, LSU	no AZA

Tab. S4, continued

Table S5: Compilation of strains of *Am. languida* obtained in this study.

Species	Strain	Origin Station	Length (µm) Mean ± SD Min-max	Width (µm) Mean ± SD Min-max	l/w ratio Mean ± SD	N	Morphological analysis	Sequence data
Am. languida	5-F11	71	**12.6**±1.2 7.4–14.9	**9.4**±0.7 7.7–11.7	**1.35**±0.10	54	LM SEM	LSU
Am. languida	6-D11	71	**12.9**±0.8 11.5–14.4	**10.5**±0.7 9.1–12.2	**1.23**±0.06	54	LM SEM	LSU
Am. languida	7-G6	71	**14.4**±1.1 12.3–16.6	**11.8**±1.0 9.8–14.5	**1.23**±0.06	51	LM SEM	LSU
Am. languida	7-H4	71	**13.3**±0.9 10.7–14.9	**10.9**±0.8 9.6–13.1	**1.22**±0.10	40	LM SEM	LSU
Am. languida	7-F8	71	**13.3**±0.7 11.6–14.9	**10.9**±0.9 9.1–14.4	**1.22**±0.09	51	LM	LSU
Am. languida	5-F10	71	**13.4**±1.3 10.7–15.8	**10.0**±1.0 7.5–12.5	**1.34**±0.10	51	LM	LSU
Am. languida	7-H5	71	**13.6**±1.1 10.4–16.0	**10.9**±1.0 8.8–13.3	**1.25**±0.08	51	LM	LSU
Am. languida	8-C4	71	-	-	-	-	LM SEM	LSU
Am. languida	8-D10	71	-	-	-	-	LM SEM	LSU
Am. languida	7-G4	71	-	-	-	-	LM	LSU

Table S6: Uncorrected p-distances of ITS sequence data between different *Az. spinosum* ribotypes, *Az.* cf. *spinosum*, and other closely related *Azadinum* species. Based on the threshold suggested by Litaker et al. [1] of 0.04 to differentiate between dinophyte species the values are highlighted as green (< 0.04), yellow (= 0.04) or red (> 0.04).

	3-B4 *spinosum* A	3D9 *spinosum* A	7-D3 *spinosum* B	H-4-G1 *spinosum* C	5-B9 cf. *spinosum*	1-H10 cf. *spinoum*	A2D11 *triniatum*	3D6 *cuneatum*	UTHC8 *poporum*	2E10 *obesum*
3-B4 Az. *spinosum* A*										
3D9 *Az. spinosum* A	0.01									
7-D3 *Az. spinosum* B*	0.02	0.03								
H-4-G1 *Az. spinosum* C	0.03	0.03	0.03							
5-B9 *Az.* cf. *spinosum**	0.06	0.07	0.06	0.07						
1-H10 *Az.* cf. *spinosum**	0.05	0.06	0.05	0.06	0.02					
A2D11 *Az. triniatum*	0.06	0.06	0.06	0.07	0.06	0.05				
3D6 *Az. cuneatum*	0.11	0.11	0.10	0.11	0.09	0.09	0.08			
UTHC8 *Az. poporum*	0.08	0.08	0.08	0.09	0.07	0.06	0.08	0.08		
2E10 *Az. obesum*	0.06	0.06	0.06	0.07	0.04	0.03	0.05	0.08	0.05	
AZCH02 *Az. dalianense*	0.09	0.09	0.09	0.10	0.10	0.09	0.09	0.11	0.09	0.08

1. Litaker, R.W.; Vandersea, M.W.; Kibler, S.R.; Reece, K.S.; Stokes, N.A.; Lutzoni, F.M.; Yonish, B.A.; West, M.A.; Black, M.N.D.; Tester, P.A. Recognizing dinoflagellate species using ITS rDNA sequences. *J. Phycol.* **2007**, *43*, 344-355.

Table S7. Specificity test of the *Az. spinosum*, *Az. poporum* and *Am. languida* qPCR assays on normed DNA concentration (1 ng µL^{-1}) of pre-existing reference strains and newly gained strains from this study (in red) by comparisons of mean C_T values (technical replicates, n=3). Extended from Wietkamp et al. (2019c). n.a. = not assigned; ND = not detected.

Species	Strain	Toxin profile	Ribotype	Result of the *Az. spinosum* assay	Result of the *Az. poporum* assay	Result of the *Am. languida* assay
Az. spinosum	3D9	AZA-1, -2, -33	A	C_T = 19.3	ND	ND
	4-F8	AZA-1	A	C_T = 18.4	ND	ND
	5-C11	AZA-1, -2, -33	A	C_T = 18.4	ND	ND
	6-G8	AZA-1, -2	A	C_T = 19.4	ND	ND
	N-04-01	AZA-1, -2, -33	A	C_T = 19.4	ND	ND
	Shet-F6	AZA-1, -2, -33	A	C_T = 19.2	ND	ND
	SM2	AZA-1, -2, -33	A	C_T = 19.8	ND	ND
	UTH-E2	AZA-1, -2, -33	A	C_T = 19.1	ND	ND
	5-F3	AZA-11, -51	B	C_T = 25.6	ND	ND
	8-B8	AZA-11, -51	B	C_T = 25.4	ND	ND
	H-1-D11	AZA-2	B	C_T = 26.1	ND	ND
	N-04-04	AZA-11, -51	B	C_T = 25.7	ND	ND
	N-05-01	AZA-11, -51	B	C_T = 26.8	ND	ND
	N-16-02	AZA-11, -51	B	C_T = 25.2	ND	ND
	H-4-A10	ND	C	ND	ND	ND
	H-4-A1	ND	C	ND	ND	ND
	H-4-C10	ND	C	ND	ND	ND
Az. cf. *spinosum*	1-H10	ND	n.a.	ND	ND	ND
	2-A3	ND	n.a.	ND	ND	ND
	5-B9	ND	n.a	ND	ND	ND
	5-D3	ND	n.a.	ND	ND	ND
	6-A1	ND	n.a.	ND	ND	ND
Am. languida	2-A11	AZA-38, -39	n.a.	ND	ND	C_T = 20.5
	AND-A0920	AZA-2, -43	n.a.	ND	ND	C_T = 20.4
	5-F11	AZA-38, -39	n.a.	ND	ND	C_T = 20.7
	8-D10	AZA-38, -39	n.a.	ND	ND	C_T = 20.4

Table S8: *Az. spinosum* ribotype A. Total AZA cell quota (fg cell^{-1}) single strains, summary statistics of multiple measurements. Toxin profile type: 1: AZA-1, -2, -33; 2: AZA-1; 3: AZA-1, -2; 4: AZA-1, -33. "period" indicates the time period (months) in which multiple measurements were performed.

strain	tox-type	median	SD	min	max	fold	n	period
2-G4	1	16.2	9.7	13.9	37.7	2.7	4	18
4-C2	1	31.6	22.8	7.4	63.1	8.5	3	16
5-E4	1	18.1	7.1	1.3	20.6	16.2	5	16
6-F8	1	12.2	5.7	3.7	18.3	4.9	4	18
2-E9	1	5.3	5.6	4.2	19.1	4.5	5	18
4-C9	2	18.9	10.9	8.0	29.8	3.7	2	16
4-D6	3	13.8	9.7	9.3	36.5	3.9	5	16
6-G8	3	4.7	6.3	1.8	19.7	11.1	5	16
2-C8	4	8.0	5.4	5.7	20.7	3.6	5	16
2-B5	1	14.8	6.8	1.2	16.2	13.6	3	8
2-E2	1	24.9	5.4	16.8	29.8	1.8	3	8
5-C11	1	17.6	19.7	9.3	58.9	6.3	4	9
6-G5	1	10.2	7.6	3.5	24.1	6.9	4	9
7-C1	1	18.1	9.4	1.6	23.9	15.0	3	9
7-E10	1	12.4	6.5	5.9	18.9	3.2	2	8
7-F4	1	8.1	4.2	1.5	11.7	8.0	3	8
3-E6	2	1.5	0.7	1.4	2.9	2.2	3	8
4-E11	2	7.3	0.6	7.1	8.3	1.2	3	8
4-F8	2	6.9	16.7	4.2	44.4	10.6	4	9
2-G10	3	10.7	4.0	9.9	18.8	1.9	3	8
4-G9	3	18.8	4.3	11.4	21.6	1.9	3	8
2-E7	3	12.1	18.9	6.6	49.1	7.5	3	8
6-A10	4	16.2	13.7	3.3	36.6	11.1	3	8

Table S9: *Az. spinosum* ribotype A. AZA-1/-2 ratio, variability single strains, multiple measurements. Toxin profile type: 1: AZA-1, -2, -33; 2: AZA-1; 3: AZA-1, -2; 4: AZA-1, -33. "period" indicates the time period (months) in which multiple measurements were performed.

strain	tox-type	median	SD	min	max	fold	n	period
2-G4	1	3.8	0.6	3.0	4.6	1.5	4	18
4-C2	1	8.9	4.5	3.8	14.7	3.9	3	16
5-E4	1	1.7	3.4	1.6	10.2	6.5	5	16
6-F8	1	4.6	1.1	3.1	6.1	2.0	4	18
2-E9	1	4.5	1.0	2.6	5.3	2.0	5	18
4-C9	2						3	16
4-D6	2	1.2	0.3	0.7	1.6	2.1	5	16
6-G8	2	1.9	0.3	1.8	2.5	1.4	5	16
2-C8	4						5	16
2-B5	1	1.8	0.4	1.2	2.1	1.7	3	8
2-E2	1	2.3	0.1	2.3	2.6	1.1	3	8
5-C11	1	1.9	0.4	1.3	2.4	1.8	4	9
6-G5	1	2.3	0.6	1.9	3.4	1.9	4	9
7-C1	1	1.3	0.4	1.1	2.0	1.8	3	9
7-E10	1	2.6	0.8	1.7	3.4	1.9	2	8
7-F4	1	1.9	0.3	1.8	2.5	1.4	3	8
3-E6	2						3	8
4-E11	2						3	8
4-F8	2						4	9
2-G10	3	10.5	2.6	5.8	12.0	2.1	3	8
4-G9	3	1.8	0.2	1.4	1.8	1.3	3	8
2-E7	3	2.7	0.3	2.1	2.9	1.4	3	8
6-A10	4						3	8

Table S10: *Az. spinosum* ribotype A. Ratio AZA-1/-33, variability single strains, multiple measurements. Toxin profile type: 1: AZA-1, -2, -33; 2: AZA-1; 3: AZA-1, -2; 4: AZA-1, -33. “period” indicates the time period (months) in which multiple measurements were performed.

strain	tox-type	median	SD	min	max	fold	n	period
2-G4	1	4.3	1.9	3.7	8.5	2.3	4	18
4-C2	1	8.0	2.0	7.4	11.9	1.6	3	16
5-E4	1	1.0	0.3	0.7	1.4	2.1	5	16
6-F8	1	7.1	2.2	2.7	8.6	3.1	4	18
2-E9	1	7.1	3.8	3.6	14.2	3.9	5	18
4-C9	2						3	16
4-D6	3						5	16
6-G8	3						5	16
2-C8	4	2.7	0.8	2.1	4.3	2.0	5	16
2-B5	1	5.3	2.6	4.5	10.4	2.3	3	8
2-E2	1	2.3	1.4	1.7	4.9	2.9	3	8
5-C11	1	5.6	1.0	4.6	7.2	1.6	4	9
6-G5	1	6.5	4.0	2.9	13.9	4.8	4	9
7-C1	1	1.8	0.4	1.6	2.6	1.6	3	9
7-E10	1	7.7	0.0	7.6	7.7	1.0	2	8
7-F4	1	5.8	1.1	3.8	6.2	1.6	3	8
3-E6	2						3	8
4-E11	2						3	8
4-F8	2						4	9
2-G10	3						3	8
4-G9	3						3	8
2-E7	3						3	8
6-A10	4	11.1	1.7	9.0	13.1	1.4	3	8

Table S11: *Az. spinosum* ribotype B. Total AZA cell quota (fg $cell^{-1}$) single strains, summary statistics of multiple measurements. Toxin profile type: AZA-11 and AZA-51. "period" indicates the time period (months) in which multiple measurements were performed.

strain	tox-type	median	SD	min	max	fold	n	period
5-F3	11-51	2.5	5.5	0.2	14.0	68.1	4	13
5-F7	11-51	1.2	0.8	0.4	2.4	6.6	4	13
5-G8	11-51	1.8	1.6	0.2	3.5	21.6	2	14
7-D3	11-51	3.1	2.6	0.1	5.6	63.7	4	13
7-E4	11-51	0.6	0.4	0.2	1.0	4.7	2	13

Table S12: *Az. spinosum* ribotype B. AZA-11/-51 ratio, variability single strains, multiple measurements. Toxin profile type: AZA-11 and AZA-51. "period" indicates the time period (months) in which multiple measurements were performed.

strain	tox-type	median	SD	min	max	fold	n	period
5-F3	11-51	1.4	0.5	0.6	1.9	2.6	4	13
5-F7	11-51	0.3	0.2	0.1	0.5	3.6	4	13
5-G8	11-51	1.1	0.7	0.3	1.8	5.3	2	14
7-D3	11-51	1.1	0.5	1.1	2.3	2.2	4	13
7-E4	11-51	0.8	0.1	0.7	0.8	1.2	2	13
